# Internal controls driven by mindfulness toward enhanced ethical behaviors: Empirical evidence from Vietnam

**DOI:** 10.1016/j.heliyon.2023.e18002

**Published:** 2023-07-06

**Authors:** Nguyen Phong Nguyen, Tu Thanh Hoai

**Affiliations:** School of Accounting, University of Economics Ho Chi Minh City, Ho Chi Minh City, Viet Nam

**Keywords:** Internal control structure, Internal control effectiveness, Organizational mindfulness, Ethical behaviors, Emerging markets, Vietnam

## Abstract

This study develops and tests a moderated mediation model regarding the effectiveness of internal control structure on organizational ethical behaviors via the mediating role of internal control effectiveness and the moderating role of organizational mindfulness in the relationship between internal control structure and internal control effectiveness. The proposed model and its hypotheses were tested using partial least squares structural equation modeling (PLS-SEM) in SmartPLS3 with survey data from 540 large Vietnamese manufacturing and service firms. This study found the following: (1) The internal control structure positively affects internal control effectiveness, which in turn promotes ethical organizational behaviors; and (2) The effect of internal control structure on internal control effectiveness is amplified by strengthening organizational mindfulness. The findings demonstrate that combining internal control systems and organizational mindfulness contributes to the ethical business practices of firms in an emerging market. Our study bridges the gap in the literature on internal control and mindfulness by providing empirical evidence on how the interaction between organizational mindfulness and internal control systems can promote ethical business practices. Additionally, our study advances the current understanding of how internal control systems can interact with organizational mindfulness to influence ethical business practices in the context of an emerging market.

## Highlights

• Internal control structures positively influence internal control effectiveness.
• Internal control effectiveness positively influences organizational ethical behavior.
• Internal control effectiveness mediates the effect of an internal control structure on ethical organizational behaviors.
• Organizational mindfulness positively moderates the effect of internal control structures on internal control effectiveness.

## 1 Introduction

The accounting literature has a wealth of information on how internal control structures affect organizational effectiveness [[1], [2], [3], [4]]. Internal control studies primarily concentrate on how internal control systems help organizations prevent and detect fraud and errors, manage risks, deal with agency problems, and ultimately promote corporate governance [[5], [6], [7], [8]]. Internal control systems refer to the set of controls and procedures formulated by a company to achieve its objectives: improving operational effectiveness and efficiency, enhancing financial reporting reliability, and ensuring compliance with laws and regulations [9].

Additionally, several studies have established that internal control systems effectively cope with ethical issues [5,7,[10], [11], [12]]. While prior research has also established a link between internal control systems and individual ethical choices [7,10,13], the understanding of how internal control systems can foster ethical behaviors at the organizational level is limited, particularly in Asian transition economies. In these economies, “good ethics is good for business” and “doing well by doing good” are common notions [14]; however, their business ethics are still considered immature compared to the developed economies in the West [15]. The concept of promoting ethical behaviors is attracting the attention of businesses in emerging Asian markets, where business ethics remain a major concern and academic research on this subject is scarce [16].

Our study addresses this research gap by introducing the concept of organizational mindfulness and examining its interaction with internal control systems to foster ethical behaviors. Mindfulness is “an enhanced attention to and awareness of a current experience or present reality” [17] that entails interpretative work directed at weak signals, differentiation of conventional wisdom, and reframing, which can help expand our understanding of observations [18]. Mindfulness is a beneficial trait for individuals because it enables them to adapt more effectively to environmental demands. It has been linked to greater executive attention in conditions that require increased self-regulation [19]. At the organizational level, mindfulness refers to a firm’s willingness and capability to pay attention to and capture discriminatory details about its settings, continuously refine and differentiate these details in light of newer experiences, and make sense of unexpected events through these details [20,21]. Given the widespread perception that mindfulness is more prevalent in Asia than in the West [22], the concept of organizational mindfulness may be relevant in the research setting of this study: Vietnam, an Asian developing country.

Studies on the interplay between mindfulness and ethics-related constructs in organizational contexts [16,[23], [24], [25], [26], [27]] reveal that mindfulness and business ethics are positively associated. For example, mindfulness promotes environmentally responsible behavior [24], relates to pro-environmental behavior and a sense of connection to nature [23], and is highly associated with moral reasoning [26,27]. Mindfulness could develop an ethical vision, a “re-description of reality,” leading to an ethical way of life [28]. Additionally, mindfulness enhances moral sensitivity, which contributes to ethical decision-making [29]. The relationship between mindfulness and ethical behaviors also manifests in various ways, such as improved awareness levels [30] and reduced moral disengagement [31]. Moreover, mindfulness enhances employees’ likelihood of acting ethically, adhering to ethical standards, and approaching ethical decision-making through a deliberate and thorough approach [32].

Although the link between mindfulness and business ethics has been documented in developing Asian countries [16,25], little is known about how internal control systems and organizational mindfulness interact to foster organizational ethical behaviors in these countries. This gap is critical because understanding this interaction may enable these firms to tailor their internal control systems and mindfulness practices to maximize operational efficiency while adhering to ethical standards under stakeholder pressure. Whereas internal controls allow firms to achieve their best organizational performance, organizational mindfulness will enable them to fulfill their societal and related responsibilities [33]. Thus, the gap in the potential interaction between internal control systems and organizational mindfulness and the effect of this interaction on ethical behaviors warrants further investigation.

Our study aimed to address two research objectives that stem from existing research gaps. First, we seek to investigate the impact of internal control structures on organizational ethical behaviors through the mediating role of internal control effectiveness. This objective is directly linked to our first research question: Do internal control structures affect ethical behaviors via the mediating role of internal control effectiveness? This research question is critical as it allows us to comprehend how internal control structures can be leveraged to promote ethical behaviors within organizations. Second, our study aimed to explore the moderating role of organizational mindfulness in the relationship between internal control structure and internal control effectiveness. This objective links to our second research question: Can organizational mindfulness strengthen the association between internal control structure and internal control effectiveness? This research question is equally crucial as ignoring the moderating impact of organizational mindfulness could result in unnecessary expenses for businesses that invest resources in developing and implementing a sophisticated internal control structure to enhance internal control effectiveness.

In bridging these gaps and achieving the research objectives, our study developed and tested a moderated mediation model explaining how internal control structures affect organizational ethical behaviors via the mediating role of internal control effectiveness and the moderating role of organizational mindfulness. In doing so, this study makes several contributions to the extant literature. First, while the literature on the interface between internal controls and business ethics [7,10,11,13] has neglected to investigate the role of internal control systems in promoting organizational ethical behaviors with the presence of mindfulness, our study provided an insight into how an internal control structure affects organizational ethical behaviors in emerging markets under the facilitating role of organizational mindfulness. Second, we validated the moderated mediation using a representative sample of Vietnamese firms. Vietnam was chosen as the research site because it is a one-party socialist republic with a less well-functioning bureaucratic structure and a highly relationship-dependent culture [27]. These characteristics resemble those of China but may differ from those of Western and other Asian developing nations. Therefore, it is interesting to understand how the interaction between internal control systems and organizational mindfulness practices can promote ethical behaviors in this country. Being the third-largest and one of the fastest-growing transitional economies [16], Vietnam is attracting more foreign direct investment (FDI); hence, improving organizational ethical behaviors allows it to attract greater FDI inflows. Therefore, testing the research model in the context of Vietnam can contribute to the limited literature on mindfulness and business ethics in emerging market contexts [16,25,34,35] by providing further insight into how internal control systems can promote ethical behaviors under the boundary condition of organizational mindfulness. This contribution is important because firms in these markets frequently lack an understanding of organizing and implementing effective internal control systems to address prevalent business ethics issues.

The remainder of this article is organized as follows: The second section presents the theoretical background underpinning our model and hypotheses. The third section discusses the research methods, such as data collection and measurement scales. Following the data analysis, results, and discussion of study findings in the fourth section, the fifth section considers the theoretical and managerial implications, limitations, and future research directions.

## 2 Theoretical background and hypothesis development

### 2.1 Internal control structure and internal control effectiveness

The Committee of Sponsoring Organizations of the Treadway Commission (COSO) defines internal control as “a process, effected by an entity’s board of directors, management, and other personnel, designed to provide reasonable assurance regarding the achievement of objectives relating to operations, reporting, and compliance” [36]. COSO’s “internal control-integrated framework (COSO framework) identifies five components of an internal control structure: the control environment, risk assessment, control activities, information and communication, and monitoring.

The COSO framework presupposes the presence and proper operation of these five components, each of which is critical to achieving a firm’s internal control goals [36]. The first component (control environment) is crucial for the subsequent components, as it establishes the tone and culture of an organization, around which all subsequent actions are built. The second component (risk assessment) ensures that essential steps are taken to address and analyze any threats to the firm’s objectives, thereby laying the foundation for risk management. The third component (control activities) is concerned with ensuring the implementation of management’s directions, specifically the policies and processes. The fourth component (information and communication) generates information regarding the dangers and changes. Finally, the fifth component (monitoring) determines the quality of an internal control system.

Internal control effectiveness refers to how well three internal control objectives are accomplished^9^. These objectives are (1) operational effectiveness and efficiency, enabling businesses to respond appropriately to risks, achieve performance and profitability targets, and protect assets against loss; (2) financial reporting reliability, encompassing trustworthy financial reports and methods for disclosing control defects and executing remedial measures; and (3) compliance with applicable laws and regulations [9,37].

The COSO framework is predicated on the assumption that all five components result in internal control effectiveness. Using empirical evidence from a sample of Finnish companies, Länsiluoto et al. [38] demonstrated that variations in internal control structure are associated with differences in control effectiveness. Effective internal control necessitates the design and operation of the five internal control components to ensure effective and efficient operations, financial reporting reliability, and compliance with laws and regulations [2,38]. Specifically, the control environment component enables a firm to establish realistic operational goals and ensures that it has adequate resources to pursue them [38]. The control activities component ensures that the firm takes the necessary steps to address the threats to achieve its operational objectives. Data regarding such threats can be identified, generated, and analyzed using risk assessment as well as information and communication components [38]. Finally, the monitoring component enables firms to ascertain whether laws and regulations have been effectively implemented [36]. The five components of the internal control structure also assist firms in reducing the risk of material errors and fraud in financial statements, ensuring the efficient utilization of resources, and enhancing organizational flexibility and the ability to coordinate between functional departments, thereby enhancing their ability to operate more effectively and efficiently [1]. Based on this reasoning, we propose the following hypothesis:

H1 Internal control structures positively influence internal control effectiveness.

### 2.2 Internal control effectiveness and organizational ethical behavior

Organizational ethical behavior is a subfield of ethics that focuses on applying moral standards to organizations [39]. The inception of organizational ethical behaviors is a code of conduct or guidance that defines what constitutes proper corporate behavior, with the accompanying goal of proper action satisfying the code of conduct [40]. Therefore, organizations must provide guidelines for employees regarding acceptable workplace practices [41]. Furthermore, as part of the control environment component of the internal control structure, managers with high levels of integrity not only implement formal policies, authority, and structure to guide the strategic orientation of the organization but also influence employees through their symbolic roles and examples of acceptable behaviors [42]. Therefore, we argue that internal control systems that provide clear guidance and fair procedures can positively affect employees’ attitudes, thereby shaping their ethical behaviors.

Whistleblowing is perhaps one of the most explored consequences in the organizational behavior and behavioral ethics literature regarding explicit positive behaviors [43,44], and promoting organizational ethical behaviors is a concern. In this context, an effective internal control system allows an organization to review, evaluate, and respond to whistleblowing and warn against potentially unethical behaviors. Such a system can assist businesses in eliminating fraud and errors, managing risks, and achieving business objectives [7]. Therefore, internal control processes and procedures can guide and monitor ethical organizational behaviors and enable businesses to comply with ethical principles and rules.

Moreover, internal control effectiveness can minimize fraudulent behaviors detrimental to organizations or prevent violations of ethical standards [7,45]. Effective internal control systems can detect and correct actions inconsistent with ethical standards [3, 5, 11]. Additionally, the marketplace is gaining more awareness of and discrimination against businesses that do not adhere to ethical business operations and management principles [46]. Therefore, ethical behavioral control systems should assess whether a particular decision advances the broader objective of a rule or regulation that controls employee behavior in an organizational context [7]. If the rules established by an internal control system are deemed appropriate, fair, and capable of preventing employee wrongdoing, the organization can promote ethical behaviors. Moreover, employees are responsible for following rules and regulations under the pressure of an effective internal control system [47], which strengthens organizational ethical behavior. On the other hand, when an organization’s internal control deteriorates, its audit functions and corporate governance also deteriorate, exposing the organization to fraud opportunities [46]; thus, employees are more likely to engage in unethical behaviors [48]. Considering the preceding arguments, the following hypotheses are proposed:

H2 Internal control effectiveness positively influences organizational ethical behavior.

H3 Internal control effectiveness mediates the effect of an internal control structure on ethical organizational behaviors.

### 2.3 The moderating role of organizational mindfulness

Organizational mindfulness refers to an organization’s ability to discriminate details about impending dangers and respond promptly to these details [49]. This term captures the collective mindfulness of an organization’s managers and employees [16]. The more organizational mindfulness an organization has, the more accountable people are for identifying and resolving challenges to the organization’s objectives. The term organizational mindfulness also refers to the degree to which an organization’s attention is focused on volatile and unexpected situations [21].

Vogus and Sutcliffe [49] claim that organizational mindfulness has strategic and operational benefits and that it occurs when leaders communicate the value of mindfulness to employees, which motivates them to act more mindfully. Hence, employees develop a greater focus on their work, which improves the effectiveness of internal control systems. Moreover, mindfulness has been proven to improve attention at work, including “concentration, sustained attention, orienting, alerting, conflict monitoring, executive processing, and behavioral inhibition,” while “cognitive distortion, reflection, suppression, and thought control” were all measured aspects of cognition [50]. Chang and Stone [50] hypothesized that mindfulness could minimize automatic processing biases, enhance mindset matching to tasks and contexts, and improve calibration by reducing overconfidence, confirmation bias, and the framing effects’ influence. By contrast, less-mindful employees may not be aware of the problems in their dilemmas [34]. Additionally, while effective internal control requires mindfulness as an awareness of early warning signs and the ability to learn from failures or mistakes, growing attention to audibility, system compliance, and reputational risk could necessitate emphasizing and allocating additional time to internal control practices [51].

Organizational mindfulness entails exercising professional judgment when deviating from standard operating procedures in unusual circumstances and engaging in informal micro-practices of framing challenges in novel ways [51]. Therefore, organizational mindfulness can exacerbate the constraints associated with implementing regular and standardized internal control systems to increase their effectiveness. Moreover, because organizational mindfulness can assist businesses in identifying and evaluating potential threats and opportunities [20,52], the likelihood of firms achieving their internal control objectives increases. Therefore, we argue that the impact of an internal control structure on internal control effectiveness becomes profound if organizational mindfulness increases. Accordingly, we propose the following hypothesis:

H4 Organizational mindfulness positively moderates the influence of internal control structures on internal control effectiveness.

**Fig. 1:**
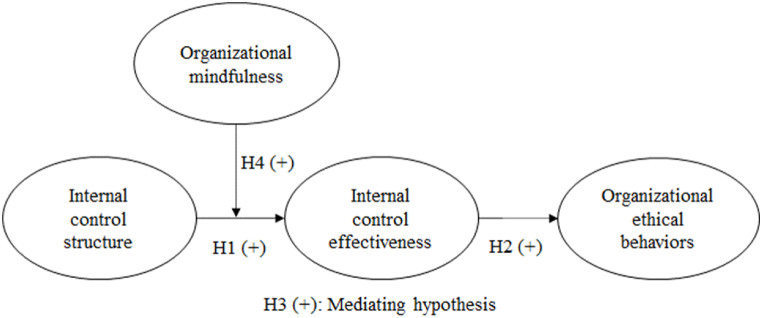
Proposed model.

Fig. 1 depicts the proposed model. The proposed model is later referred to as Model 3 in Table 4 of this article.

## 3 Research methods

### 3.1 Research site

The research setting for this study was Vietnam, an Asian transitional economy. Since the Renovation Program (Doi Moi) in 1986, Vietnam’s global trade policy has rapidly changed. With an average annual gross domestic product (GDP) growth rate of 6.6%, Vietnam is among the top 50 economies globally [53]. For the following two reasons, it is the ideal setting to investigate the relationships among mindfulness, internal control, and business ethics.

First, thorough knowledge of ethical business practices in Vietnam is crucial because, although it is one of the fastest-developing transition economies, it is plagued by extensive business ethics problems [16], Vietnam’s corporate environment is fraught with ethical issues and dilemmas, as there is no unambiguous moral right to guide decision-making. Thus, while a manager’s strong moral character is critical, such a character must be mindfully handled with complex contemporary managerial issues [27]. Therefore, examining how organizational mindfulness contributes to the development of internal control systems to promote business ethics in Vietnam can add to the limited research on the interface between internal control and business ethics in an emerging market context.

Second, while the Western world has substantially researched ethics, little is known about Vietnamese culture, commercial practices, or ethical norms [47]. Third, business ethics is a relatively recent notion in Vietnam, despite the early introduction of moral principles, concepts, and lessons. For many Vietnamese, morality and ethics are merely about cooperating with and obeying higher authorities (power distance). Through the Renovation Program, the Vietnamese government tried to protect consumer rights and intellectual property; enhance the quality of products, services, and safety; and promote corporate social responsibility. However, many still equate ethics with following rules and laws [47].

### 3.2 Data collection

We surveyed large Vietnamese manufacturing and service business organizations in mid-2020. Given that firms in these industries are more likely to face ethical dilemmas than those in the trading industry, our choice was consistent with other business ethics studies in Asia [14,16]. To guarantee the quality of the survey, we selected respondents who satisfied the following requirements: (1) at least two years of experience in their company; (2) mid- and top-level managers in large Vietnamese business organizations with an internal control department; and (3) background in accounting, finance, sales, marketing, production, or research and development, as these departments are closely linked to internal control. The list of target informant emails, comprising 4.258 contacts, was compiled using information gleaned from LinkedIn, a social networking site for professionals. According to Mintz and Currim [54], LinkedIn can be used to gather emails from prospective responders. Furthermore, we discovered that sending survey questionnaires via LinkedIn emails helped us contact many managers across the country more conveniently, whereas sending questionnaires via post or in person could endanger the research participants owing to the COVID-19 pandemic.

The survey questionnaire was framed on the basis of the measurement scales of the main constructs. It was prepared in English and then translated into Vietnamese by bilingual scholars using Brislin’s back-translation process [55]. The questionnaire was again back-translated from Vietnamese into English to avoid translation problems. Additionally, the Vietnamese version was piloted with five managers and fine-tuned in response to their suggestions. The procedures stated above helped ensure the applicability of the content validity survey questionnaire in the Vietnamese context. Moreover, we obtained informed consent from all participants, and because we did not require their identities or the names of their companies, complete confidentiality and anonymity were guaranteed.

We collected data in two stages to avoid common method bias. As Einarsen et al. [56] recommended, we employed a two-month interval between stages to avoid high dropout rates and memory bias. In Stage 1, we sent the survey questionnaire to 4,258 potential informants and received 989 completed responses, yielding a response rate of 23.23% [989/4.258]. Their responses reflected their demographic information and understanding of their organizations’ internal control structures and organizational mindfulness. The data collection in Stage 2 involved sending the second part of the survey to the respondents of Stage 1, which was used to collect information about the effectiveness of internal controls and organizational ethical behaviors. Each participant was assigned an identification number as a reference for the two stages of data collection. After Stage 2, we obtained 540 valid responses from our final sample, with a final response rate of 12.68% [540/4,258]. This response rate was acceptable and comparable to previous studies using email surveys in Vietnam [16,37].

As the study was conducted at the organizational level, we carefully scanned the sample for possible duplicate responses from the same organization. The scanning procedure included verifying company information (e.g., company name, business email address, and domain name) to ensure that each firm in the sample provided a single response. No such occurrences were discovered during this process. Table 1 summarizes the demographic characteristics of the participating businesses and informants. As a homogeneous sample of the current study may have been lacking when we collected data from a mix of managers at the top (29.26%) and middle levels (70.74%) from diverging subsectors in the manufacturing (40.93%) and services (59.07%) industries, the internal validity of the study’s results can be affected. We then performed independent t-tests across different industries (i.e., manufacturing and services) and informants (i.e., top managers and mid-level managers) and found no significant differences between these groups in terms of the main variables in the proposed model. Therefore, the measurement model was homogeneous and had the necessary validity and reliability to test the hypotheses of the proposed structural model.

**Table 1:** Demographics of the participating firms and informants (*n* = 540).

Demographics	*N*	%	Demographics	*n*	%
*Job position*			*Ownership structure*		
Top-level managers	158	29.26	With foreign capital	374	69.26
Mid-level managers	382	70.74	Without foreign capital	166	30.74
*Organizational tenure (years)*			Type of ownership		
2–5	257	47.59	100% foreign-owned enterprise	233	43.15
6–10	165	30.56	SOEs (≥51% states capital)	37	6.85
11–20	96	17.78	Private company	142	26.30
>20	22	4.07	Joint venture	81	15.00
*Industry type*			Others	47	8.70
Manufacturing	221	40.93	*Firm size (assets in VND billion)*		
Services	319	59.07	≤10	11	2.04
*Manufacturing and services sub-sector*			11–50	14	2.59
Agriculture, forestry, fisheries, mining	29	5.37	51–100	31	5.74
Processing industry, manufacturing	96	17.78	101–200	43	7.96
Electricity, water, and waste disposal	9	1.67	201–500	69	12.78
Construction	36	6.67	501–1,000	105	19.44
Wholesale and retail	16	2.96	>1,000	267	49.44
Transport, warehouse	17	3.15	*Firm size (full-time equivalent employees)*		
Hotel, restaurant	42	7.78	≤50	17	3.15
Information and communication	39	7.22	51–100	32	5.93
Financial, banking, insurance, real estate	90	16.67	101–300	123	22.78
Consulting (e.g., law, architecture)	29	5.37	301–1,000	154	28.52
Education and training	9	1.67	1,001–5,000	132	24.44
Health and social aid	21	3.89	5,001–10,000	42	7.78
Arts, entertainment, and recreation	9	1.67	>10,000	40	7.41
Others	98	18.15			

### 3.3 Measurement scales

Well-established scales from the literature were used to measure the main variables of the proposed model. Specifically, internal control structures were assessed based on the formative Likert scale proposed by Jokipii [2], with 25 items on the five dimensions of an internal control structure (control environment, risk assessment, control activities, information and communication, and monitoring) derived from the COSO framework. This formative scale has also been used in recent studies in emerging market contexts [57]. Additionally, internal control effectiveness was determined based on the subjective judgments of the managers using 12 reflective Likert scale items adapted from Hunziker^1^, which asked them to rate their confidence in the three internal control objectives (operational efficiency and effectiveness, financial reporting reliability, and compliance with applicable laws and regulations). These scales for internal control systems (i.e., internal control structure and effectiveness) were relevant because they clearly reflected the components and objectives of internal control systems under the COSO framework. The scale for organizational mindfulness was also reflective and comprised eight items developed by Valentine et al. [19]. Finally, ethical organizational behaviors were rated using a reflective Likert scale adapted from Wu et al. [58]. These scales for organizational mindfulness and organizational ethical behaviors are well-established and have been employed in various business ethics studies in the context of emerging markets [16,42]. Furthermore, following previous studies [16], we used foreign ownership (0 = without foreign capital, 1 = with foreign capital), organizational size in terms of assets and full-time equivalent employees, and organizational age (number of years since the organization’s establishment) as the control variables of organizational ethical behaviors. Table 2 presents the scales for the main variables.

**Table 2:** Scale items and latent variable evaluation.

Construct and items	Weight/Loading	*t*-test
**Internal control structure** [2]		
Control environment (formative scale)*		
The governing body/board genuinely called management’s decisions into question and evinced realizable alternatives	0.27	4.01
Managers and management have not been overworked	0.06	1.12
There has been a great deal of variation in control and management tasks	0.23	2.97
The personnel understand the content and responsibilities of their tasks	0.14	1.60
The personnel have demonstrated commitment to honesty and the ethical values of the company through their conduct	0.54	6.34
Risk assessment (formative scale)*		
The goals for the company’s operations had credible and, in my opinion, reasonable measures	0.31	3.09
Management actively evaluated both internal and external risks likely to prevent the achievement of goals	0.27	1.88
A risk analysis covering the entire company was carried out during the last year	0.17	1.43
Those in managerial functions were aware of the risks of their areas of responsibility and knew how risk management was implemented	0.35	3.03
In my opinion, the company’s risk analysis and means of protection could have been more efficient *(Reversed code)*	0.03	0.52
Control activities (formative scale)*		
Functioning controls in the company’s processes warned whenever something exceptional occurred	0.35	4.97
As soon as something exceptional and undesired was noticed, it was promptly and appropriately dealt with	0.26	3.55
In the definition of tasks, special attention was paid to authorization and the special demands of tasks	0.40	5.95
In my opinion, the internal control measures should have been stepped up still further *(Reversed code)*	(0.11)	2.58
The entire personnel had updated job descriptions	0.24	3.60
Information and communication (formative scale)*		
The personnel had no problems obtaining information about their own work tasks	(0.14)	1.64
The reports forwarded to management were sufficiently clear and contained relevant information from the management perspective	0.44	3.59
Sufficient information moved between the different divisions of the company so that the smooth, uninterrupted running of operations could be ensured (e.g., from sales to manufacturing)	0.29	2.35
Our company’s information and communications system was not quite up to date with respect to functions	(0.02)	0.46
The work was efficiently coordinated within the function and also with other functions	0.50	5.08
Monitoring (formative scale)*		
The operative information used in management was specified to the systems information of financial management	0.11	0.94
Line managers take excellent care of day-to-day control	0.35	2.94
There is active control of how the personnel obey the operating instructions issued	(0.18)	1.34
We conducted analyses-based (customer satisfaction, job satisfaction, efficiency) changes during the last year	0.34	3.38
Management has not in the last year requested accounts of the accomplishment of control measures	0.49	3.25
**Internal control effectiveness** [1]		
*Operation (CR* = *0.89; AVE* = *0.66)*		
Risks of operations are reliably controlled	0.80	42.61
Operational objectives have been achieved in recent years	0.75	28.11
Operations are characterized by high efficiency	0.86	62.92
Control activities support the achievement of operational objectives	0.84	47.01
*Financial reporting (CR* = *0.84; AVE* = *0.57)*		
In recent years, all finance-related risks were identified in due time	0.78	35.88
I am very satisfied with the effectiveness of the control activities	0.74	31.73
I have full confidence in our financial reporting	0.78	31.42
The risk of a material misstatement in the financial statements is nearly impossible	0.73	23.42
*Compliance (CR* = *0.88; AVE* = *0.64)*		
Internal guidelines are complied with	0.75	34.26
Violations of laws and standards very seldom occur	0.82	54.09
Risks associated with non-compliance are at an acceptable level	0.86	73.77
I can assure that our company complies with relevant laws and standards	0.77	29.41
**Organizational mindfulness** [19] *(CR* = *0.90; AVE* = *0.53)*		
Our company has an organization-wide sense of susceptibility to the unexpected	0.82	32.50
Everyone in our company feels accountable for the reliability	0.65	15.57
Our leaders pay as much attention to managing unexpected issues as they do to achieving formal organizational goals	0.72	24.02
People at all levels of our organization value the quality of their works	0.71	23.46
We spend time identifying how our activities potentially harm our organization, employees, our customer, other interested parties, and the environment at large	0.78	36.47
We pay attention to when and why our employees, customers, or other interested parties might feel peeved or disenfranchised from our organization	0.70	21.43
There is widespread agreement among the members on what we don’t want to go wrong	0.73	24.21
There is widespread agreement among the members about how things could go wrong	0.71	21.70
**Organizational ethical behaviors** [58] *(CR* = *0.95; AVE* = *0.68)*		
Top managers of our company regularly show that they really care about ethics	0.76	41.02
Top managers of our company represent high ethical standards	0.73	34.16
Top managers of our company guide decision-making in an ethical direction	0.74	37.43
Management in our company disciplines unethical behaviors when they occur	0.76	40.27
Employees in our company accept organizational rules and procedures regarding ethical behaviors	0.76	43.47
Organizational rules and procedures regarding ethical behavior serve only to maintain our company's public image *(Reversed code)*	0.74	35.80
Penalties for unethical behaviors are strictly enforced in our company	0.72	36.36
Ethical behaviors are the norm in our company	0.74	35.79
Ethical behaviors are rewarded in our company	0.73	36.17

**Notes:** CR: composite reliability; AVE: Average variance extracted; *: CR and AVE are not applicable for formative constructs.

## 4 Data analysis and results

### 4.1 Reliability and validity tests

Following Sarstedt et al. [59], we applied the standard model evaluation criteria to the measurement models for lower-order constructs. Additionally, we used repeated indicators to define the exogenous construct, which is an internal control structure, to minimize parameter bias in the measurement model relationships of the higher-order construct [59].

To assess the overall measurement model, we first tested the reliability and validity of the reflective latent variables using SmartPLS3. The indices of the measurement model are presented in Table 2, Table 3, respectively. As the composite reliability values of the reflective latent variables ranged between 0.84 and 0.95, these scales were highly reliable. Subsequently, we then determined the convergent validity of the indicators and the average variance extracted (AVE) values using their outer loadings. Table 2 indicates that the loadings of all scale items of the reflective latent constructs ranged from 0.65 to 0.86 and exceeded the 0.50 cutoff value [60]. All corresponding t-values of the scale items of the reflective latent variables were satisfactory as well as statistically significant and ranged between 15.57 and 73.77. Additionally, the AVE values for all the reflective latent variables exceeded 0.50 (ranging from 0.53 to 0.74). Therefore, all scales demonstrated convergent validity [61].

**Table 3:** Analysis of discriminant validity.

	1___	2___	3___	4___	5___	6___	7___	8___	9___	10___
1. Control environment	**N/A**									
2. Risk assessment	0.81**	**N/A**								
3. Control activities	0.71**	0.75**	**N/A**							
4. Information and communication	0.74**	0.75**	0.74**	**N/A**						
5. Monitoring	0.78**	0.78**	0.77**	0.86**	**N/A**					
6. Operation	0.35**	0.38**	0.33**	0.44**	0.46**	**0.81**				
7. Financial reporting	0.24**	0.27**	0.26**	0.31**	0.32**	0.69**	**0.76**			
						*0.86*				
8. Compliance	0.21**	0.19**	0.17**	0.20**	0.25**	0.58**	0.54**	**0.80**		
						*0.70*	*0.70*			
9. Organizational mindfulness	0.02	0.01	0.02	0.09*	0.05	0.19**	0.19**	0.22**	**0.73**	
						*0.21*	*0.23*	*0.26*		
10. Organizational ethical behaviors	0.15**	0.19**	0.17**	0.20**	0.18**	0.42**	0.38**	0.35**	0.08*	**0.74**
						*0.48*	*0.46*	*0.41*	*0.10*	

**Notes***:* Bold diagonal number: square root of AVE; **N/A**: AVE is not applicable for formative constructs; 1st value = Bootstrapped correlation between variables (off-diagonal); 2nd value (italic) = Heterotrait-Monotrait ratio; *, **: correlations significant at 5% and 1% levels respectively (two-tailed *t*-test).

**Table 4:** Hypothesis testing results.

		Model 1	Model 2 (ICE as the mediator)		Model 3 (ICE as the mediator and OM as the moderator)	
*Hypothesis*	*Dependent variable*	OEB	ICE	OEB	ICE	OEB
	*Independent variable*					
H1	ICS	0.24 (5.00)^c^	0.43 (12.24)^c^	0.06 (0.95)	0.42 (11.41)^c^	0.05 (0.97)
	OM				0.21 (5.37)^c^	
H4	ICS × OM				0.18 (4.71)^c^	
H2	ICE			0.44 (11.18)^c^		0.44 (11.91)^c^
	*Control variable*					
	Ownership	0.10 (2.20)^b^		0.09 (2.39)^b^		0.09 (2.40)^b^
	Size (assets)	0.09 (1.92)^a^		0.08 (1.92)^a^		0.08 (1.90)^a^
	Size (employees)	0.04 (0.91)		0.07 (1.56)		0.07 (1.60)
	Firm age	0.11 (2.99)^c^		0.11 (3.01)^c^		0.11 (3.35)^c^
	Adjusted *R*^*2*^	0.09	0.18	0.24	0.25	0.24
	*Indirect effect*			Estimate	LLCI	ULCI
H3	ICS → ICE → OEB		0.19	0.14 (7.81)^c^	0.24	

**Notes***:* ICS: internal control structure; ICE: internal control effectiveness; OM: organizational mindfulness; OEB: organizational ethical behavior; ICS × OM: the interaction between ICS and OM; LLCI, ULCI: lower- and upper-confidence interval levels, respectively; numbers in brackets: *t*-values; ^a^, ^b^, ^c^: significance at 10%, 5%, and 1% levels, respectively (2-tailed *t*-test).

Following Fornell and Larcker [62], we assessed the discriminant validity of the measurements. Table 3 illustrates that the square root of the AVE for each of the main constructs (ranging between 0.73 and 0.81) was significantly higher than the corresponding bootstrapped correlations between this construct and the others (ranging between 0.08 and 0.69), indicating a high level of discriminant validity. Moreover, we employed the heterotrait-monotrait (HTMT) test [63], which is more stringent than Fornell and Larcker criterion (1981), to further assess discriminant validity. According to Table 3, the HTMT values ranged from 0.10 to 0.86 and were significantly less than one, signifying discriminant validity. Therefore, the measurement scales used in this study had satisfactory discriminant validity.

Finally, the bivariate correlations between the main constructs in our study (see Table 3) suggest that the five dimensions of the internal control structure and the three dimensions of internal control effectiveness were positively and significantly related to each other (r = [0.17; 0.86], p < 0.01). This result corroborates our hypothesized strong association between internal control structure and internal control effectiveness. Additionally, organizational mindfulness was positively correlated with the three dimensions of internal control effectiveness (r = [0.34; 0.42], p < 0.01), implying that increased organizational mindfulness may strengthen the operational, financial, and compliance aspects of internal control effectiveness. Finally, a weak positive but statistically significant correlation between organizational mindfulness and organizational ethical behaviors (r = 0.08, p < 0.05) provides evidence that ethical behaviors can be facilitated by mindfulness, which is consistent with previous research [32,50].

### 4.2 Common method bias

Despite using a two-wave survey, when all constructs were assessed using a key informant approach, common method bias was possible [64]. Therefore, we followed Lindell and Whitney [65] and employed the marker variable technique to test for the common method bias. We asked the informants a question (“Would you prefer to visit Ha Long Bay during the national holiday this year?”), which was then utilized as a marker variable. After removing the effect of rM, the mean change in the correlations between the main constructs (rU–rA) was 0.03. The criteria mentioned above demonstrate that this study was free of common method bias. We also investigated the possibility of multicollinearity. The highest inner variance inflation (VIF) value was only 5.12, which was significantly smaller than the rule-of-thumb value of ten [66]. Therefore, multicollinearity in this study was minimal.

### 4.3 Hypothesis testing results

We ran three hierarchical models to test the proposed model and its hypotheses. Model 1 depicts the direct relationship between internal control structures and ethical organizational behavior. In Model 2, internal control effectiveness is introduced as the mediating variable in this relationship. Model 3 is the proposed model, which builds on Model 2 by including organizational mindfulness as a moderator in the relationship between internal control structure and internal control effectiveness. The relationships between these three hierarchical models and the hypotheses in the proposed model are as follows: H1 was tested using Model 1, H2 was tested using Model 2, H3 was tested using Models 1 and 2, and H4 was tested using Model 3.

The proposed model and its hypotheses were tested using Partial Least Squares Structural Equation Modeling (PLS-SEM) with SmartPLS3. PLS-SEM was adopted because of its ability to test complex models with both moderating and mediating hypotheses and is especially applied to measurement models with formative constructs and indicators [67].

Table 4 presents the indices used to determine the predictive strength of the individual route (β coefficients, t-values) paths in the structural models, and the R^2^ (squared multiple correlations) for the dependent variables (internal control effectiveness and organizational ethical behavior). These values were computed using the Partial Least Squares (PLS) algorithm and PLS bootstrapping in SmartPLS3. Most of the adjusted R^2^ values were higher than 0.10, indicating that the proposed research model fit the collected data relatively well [68]. Moreover, the SRMR value of 0.08 was less than the cutoff value of 0.09, signifying a good model fit [69].

H1 posits that an internal control structure positively influences internal control effectiveness. This hypothesis is supported by the positive and significant association between these two variables (Model 2: β = 0.43, t-value = 12.24). Additionally, our data analysis confirmed H2 regarding the positive effect of internal control effectiveness on organizational ethical behaviors (Model 2: β = 0.44, t-value = 11.18; Model 3: β = 0.44, t-value = 11.91).

To test H3, we examined the indirect effect of an internal control structure on organizational ethical behaviors via internal control effectiveness and found that it was significant (β = 0.19, 95% confidence interval = [0.14, 0.24]). This result indicated that the mediating role of internal control effectiveness connected internal control structures and ethical organizational behavior, which supported H3. Moreover, when the internal control effectiveness variable was added as the mediator in the path between internal control structure and organizational ethical behavior, the path turned from significant (Model 1: β = 0.24, t-value = 5.00) to insignificant (Model 2: β = 0.06, t-value = 0.95). This result reveals that internal control effectiveness mediates the effect of internal control structure on ethical behavior, confirming H3.

**Fig. 2:**
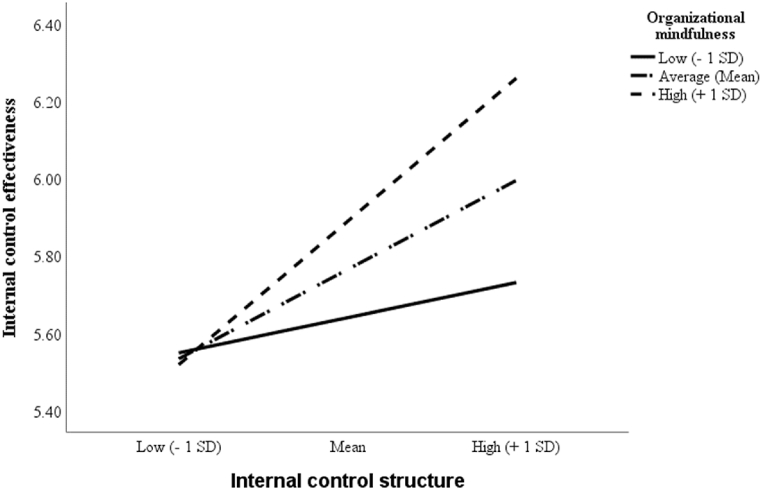
Interaction effect of internal control structure with organizational mindfulness on internal control effectiveness.

To test H4 regarding the moderating effect of organizational mindfulness on the relationship between an internal control structure and internal control effectiveness, we created an interaction term, which is the product of the independent variable, that is, internal control structure (ICS), the moderating variable, and organizational mindfulness (OM), after mean centering them to avoid multicollinearity issues [70]. As the correlation between the interaction term (i.e., ICS × OM) and internal control effectiveness was positive and significant (Model 3: β = 0.18, t-value = 4.71), this hypothesis was supported. To demonstrate the nature of this significant interaction, the effect of internal control structure on internal control effectiveness was plotted using the Johnson-Neyman approach [71] for low (−1 standard deviation), medium (mean), and high (+1 standard deviation) organizational mindfulness levels. According to Fig. 2, the impact of the level of adequacy of the internal control structure on internal control effectiveness is greater for firms with a high level of organizational mindfulness than for those with a medium or low level.

We also analyzed the effects of the control variables on ethical organizational behavior. Table 1 reveals that foreign ownership positively correlates with organizational ethical behaviors (Model 3: β = 0.09, t-value = 2.40), which implies that foreign firms adhere more frequently to higher social responsibility and ethical standards than their domestic competitors do [72]. Moreover, both firm age (Model 3: β = 0.11, t-value = 3.35), and size in terms of assets (Model 3: β = 0.08, t-value = 1.90), positively affect organizational ethical behavior, implying that large, well-established firms have more resources to implement higher ethical standards than small, newly established firms.

### 4.4 Discussion of research results

Our study examined (1) how an internal control structure affects organizational ethical behaviors via the mediating role of internal control effectiveness and (2) how organizational mindfulness moderates the effect of an internal control structure on internal control effectiveness. The first finding is that internal control structures with adequate components can improve operational effectiveness and efficiency, financial reporting reliability, and compliance with the laws and regulations of firms in Vietnam, an emerging market. This finding is consistent with those of previous studies in developed countries [1,2,38]. However, our study adds to these studies by demonstrating the importance of organizational mindfulness as a boundary condition that strengthens the positive effect of internal control structures on internal control effectiveness.

The second finding posits that internal control effectiveness mediates the relationship between internal control structure and ethical organizational behavior. More precisely, the confirmation of this mediating mechanism demonstrates that an adequate internal control structure can assist firms in an emerging market in enhancing their internal control effectiveness to effectively deal with ethical issues. This finding sheds additional light on the relationship between internal control systems and business ethics demonstrated in previous studies [7,[10], [11], [12],^73^], specifically by uncovering the path through which internal control structures positively influence ethical behaviors via internal control effectiveness in the context of emerging markets.

The third finding (i.e., organizational mindfulness moderates the relationship between internal control structure and internal control effectiveness) elucidates the conditions for an effective internal control structure. As the information on the interaction between organizational mindfulness and internal control structures was previously unknown, this finding is novel. If firms in emerging markets devote more attention to internal control activities by promoting organizational mindfulness, internal control effectiveness can be increased, especially through a well-organized internal control structure. This result agrees with previous studies (e.g., Jordan and Johannessen [51]) that claimed that effective internal control requires mindfulness as an awareness of early warning signs. This result also indicates that organizational mindfulness is effective, necessary, and noteworthy in catalyzing the management systems of organizations by promoting internal control effectiveness.

## 5 Implications, limitations, and future research directions

### 5.1 Theoretical implications

Our study makes several contributions and has theoretical implications. First, it adds to our knowledge of the role of internal control systems in facilitating ethical organizational behavior. Although the traditional literature on internal control and business ethics mentions the importance of integrating effective internal control systems into a structural and formal ethics program to guide organizational ethical behaviors [73,74], little is known about its application in the context of emerging markets. In bridging this gap, we discovered that an internal control structure is positively correlated with internal control effectiveness and affects organizational ethical conduct. These findings extend previous research on business ethics in emerging Asian markets [14,16] by positing that organizational mindfulness can act in conjunction with internal control structures to help firms in emerging markets to improve their internal control effectiveness. Therefore, we present a more nuanced picture of how internal control influences ethical organizational behaviors when organizational mindfulness acts as a moderator.

Second, this study provides a greater understanding of the conditions under which an internal control structure effectively attains operational, financial, and compliance objectives. Specifically, it adds to the limited work on how an internal control structure achieves these objectives [2,42] by exploring the moderating effect of organizational mindfulness on the relationship between internal control structure and internal control effectiveness. To the best of our knowledge, this is the first study to empirically examine organizational mindfulness and the ethical implications of internal control systems in an emerging market context.

Third, our study broadens the scholarly understanding of how internal control systems and mindfulness might contribute to ethical corporate practices outside the context of advanced economies by empirically testing our model using data from the developing Vietnamese nation. Finally, our study responds to Chang and Stone’s [50] challenge of exploring the potential benefits of mindfulness in certain facets of accounting practice. Specifically, by proving that organizational mindfulness positively moderates the link between internal control structure and internal control effectiveness, our study adds more insight into the conditions under which internal control effectively promotes the ethical behaviors of firms in emerging markets.

### 5.2 Managerial implications

In addition to the aforementioned theoretical implications, our study has several managerial implications. First, the findings that an internal control structure improves internal control effectiveness and that this relationship is significantly strengthened under elevated levels of organizational mindfulness are essential for Vietnamese firms. These findings may be generalized to other emerging Asian economies with similar business cultures, such as China and Taiwan [75,76]. The significance of this finding lies in its implication for Vietnamese managers. They could potentially promote ethical conduct by enhancing the effectiveness of their internal control systems. One way to achieve this is by developing robust accounting information systems (AIS), which can create a favorable environment for effective internal control systems [77]. This is due to the fact that AIS includes controls over the functioning of both people and systems in order to improve the effectiveness and efficiency of organizations and protect assets [78]. Moreover, AIS serves as the fundamental basis for creating precise and prompt accounting information, which, in turn, sets up a robust internal control mechanism [79]. Thus, by developing robust AIS, firms can enhance their ability to promote and maintain effective internal controls. Second, the results of this study have implications for comprehending the outcomes of an internal control structure that can help firms develop organizational ethical conduct that contributes to their corporate reputation. Overall, the critical role of the interplay between mindfulness and internal control systems in regulating business ethics suggests that this study can contribute to our theoretical knowledge and lead to managerial implications for other rapidly changing economies.

### 5.3 Limitations and future research

Despite these significant contributions, this study has several limitations that are worth highlighting. First, as this study was cross-sectional and conducted during the lockdown in Vietnam, causal relationships among internal control structures, organizational mindfulness, internal control effectiveness, and organizational ethical behaviors could have been overlooked, even if a two-wave survey approach had been employed. A follow-up longitudinal study in the post-COVID-19 era would corroborate these associations.

Second, because this study was conducted in Vietnam, it may have limited cross-national applications. Therefore, from the perspective of institutional theory, additional research should be conducted in other emerging countries with unique contextual elements (e.g., culture, legislation, ethical standards, and other religious contexts such as Christian, Jewish, or Islamic) that could provide additional insights and inform theory development. Future studies should validate the proposed model by examining the possible influence of distinct local contextual factors on large manufacturing and service enterprises in other emerging economies.

Third, as objective information on actual organizational ethical behaviors was difficult to gather using the survey approach, we had to measure this variable using the perceptions of managers in our quantitative study, as in previous studies (e.g., Cortes-Mejia et al. [42]; Nguyen et al. [16]). Thus, future research should employ qualitative methods (e.g., interviews, focus groups, and field observations) to investigate actual ethical organizational behaviors resulting from internal control systems.

Finally, we analyzed organizational ethical behaviors by controlling for the following four variables: ownership structure, firm size in terms of assets, firm size in terms of the number of employees, and firm age. Therefore, subsequent studies should incorporate other control variables (e.g., stakeholder pressure and competition intensity) that may influence ethical organizational behavior.

## 6 Conclusion

This study examined the process by which internal controls affect organizational ethical behaviors and the moderating role of organizational mindfulness in this process. By developing and testing a moderated mediation model against data from 540 large Vietnamese manufacturing and service firms, this study makes a significant contribution to the internal control literature, as it is one of the first to demonstrate the mechanism by which internal control systems promote the ethical behaviors of firms in emerging markets. In addition, this study contributes to the mindfulness literature by confirming organizational mindfulness as the boundary condition of the relationship between internal control structure and internal control effectiveness. In other words, the greater the level of organizational mindfulness, the greater the capacity of the internal control structure to improve ethical organizational behaviors through internal control effectiveness. Overall, the findings of this study encourage additional research on how integrating internal control systems and organizational mindfulness can help firms in developing economies improve their ethical behaviors.

## Author contribution statement

Nguyen Phong Nguyen; Tu Thanh Hoai: Conceived and designed the experiments; Performed the experiments; Analyzed and interpreted the data; Contributed reagents, materials, analysis tools or data; Wrote the paper.

## Data availability statement

Data will be made available on request.

## Declaration of competing interest

The authors declare the following financial interests/personal relationships which may be considered as potential competing interests:

This study was funded by the 10.13039/501100005645Ministry of Education and Training of Vietnam under Grant number B2020-KSA-01. This study was also financially supported by the 10.13039/100019455University of Economics Ho Chi Minh City.
